# Protocol for analyzing transforming growth factor β signaling in dextran-sulfate-sodium-induced colitic mice using flow cytometry and western blotting

**DOI:** 10.1016/j.xpro.2023.102249

**Published:** 2023-04-25

**Authors:** Mai Fujiwara, Lucien Garo, Amrendra K. Ajay, Alkeiver S. Cannon, Panagiota Kolypetri, Shivnarayan Dhuppar, Gopal Murugaiyan

**Affiliations:** 1 Ann Romney Center for Neurologic Diseases, Department of Neurology, Brigham and Women’s Hospital and Harvard Medical School, Boston, MA 02115, USA; 2 Boston University School of Medicine, Boston, MA 02118, USA; 3 Renal Division, Department of Medicine, Brigham and Women’s Hospital and Harvard Medical School, Boston, MA 02115, USA

**Keywords:** Cell Biology, Cell Isolation, Flow Cytometry/Mass Cytometry, Immunology, Model Organisms, Molecular Biology, Signal Transduction

## Abstract

Transforming growth factor β (TGF-β) is critical to the maintenance of intestinal immune homeostasis. Here, we present techniques for analyzing Smad molecules downstream of TGF-β receptor signaling in dextran-sulfate-sodium-induced colitic mice. We describe colitis induction, cell isolation, and flow cytometric cell sorting of dendritic cells and T cells. We then detail intracellular staining of phosphorylated Smad2/3 and western blotting analysis of Smad7. This protocol can be performed on a limited number of cells from many sources.

For complete details on the use and execution of this protocol, please refer to Garo et al.^1^

## Highlights

• DSS-induced colitis in mice and colonic lamina propria mononuclear cell isolation
• Flow cytometric cell sorting for colonic lamina propria DCs and CD4+ T cells
• Intracellular staining for phosphorylated Smad2/3 in DCs and T cells
• Western blotting analysis of Smad7 in DCs and T cells

## Abstract (editor highlights)

Publisher’s note: Undertaking any experimental protocol requires adherence to local institutional guidelines for laboratory safety and ethics.

## Graphical abstract

**Figure:**
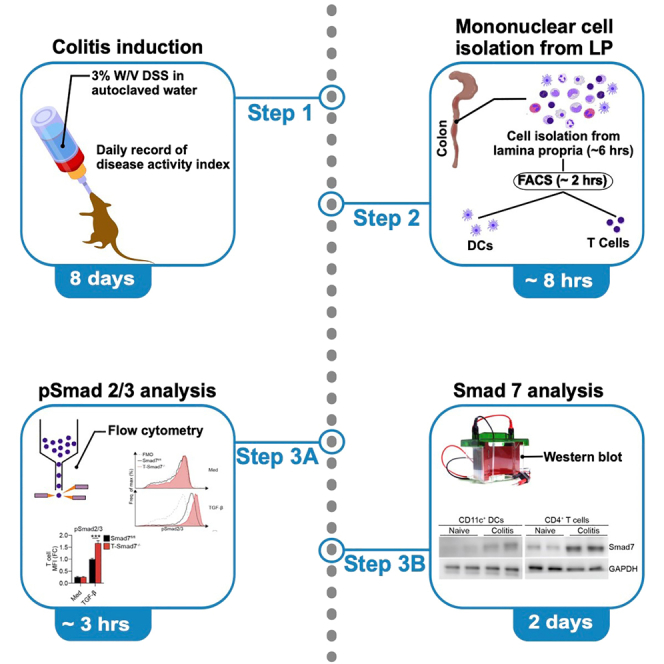

## Before you begin

Dysregulation of TGF-β signaling is associated with several inflammatory disorders including intestinal inflammation in animal models and inflammatory bowel diseases (IBD) in humans.^2^^,^^3^ The amplitude of TGF-β signaling is tightly regulated by Smad proteins.^2^^,^^3^ While Smad7 limits TGF-β signaling, Smad2 and Smad3 promote TGF-β signaling and prevent tissue inflammation and IBD.^2^^,^^3^ Below, we describe a detailed protocol for the analysis of Smad molecules. The phospho flow cytometry approach we describe can be applied to detect a variety of phospho proteins in other signaling pathways at the cellular level with even a small number of cells as compared to time-consuming traditional approaches requiring higher cell numbers.^2^^,^^4^ Further, these methods can also be applied to a variety of cell lines and primary cells such as peripheral blood mononuclear cells (PBMCs).

### Preparation of solutions and equipment

**Timing: 1 h**

On the day before the experiment.

1. Prepare buffers A, B, and C. Keep at 4°C for up to 2 weeks. For 20 mice, make 1500 mL of Buffer A, 500 mL of Buffer B, and 500 mL of Buffer C.
2. Label 100 × 15 mm style petri dishes, sterile 50 mL centrifuge tubes (five tubes per mouse), sterile 25cm^2^ vented cap, TC treated tissue culture flasks (two per mouse), and 2 mL Eppendorf tubes (one per mouse).
3. Place 100 μm cell strainer on one 50 mL conical tube per mouse and a 40 μm cell strainer on one 50 mL conical tube per mouse.

On the day of the experiment, before euthanization.

4. Set the incubator shaker at 37°C.
5. Add 0.077 g DTT to Buffer C and warm up 10 mL of Buffer C per mouse at 37°C.
6. Warm up 10 mL per mouse of the X-VIVO 15 Serum-Free medium with Gentamicin and Phenol Red at 37°C.
7. Fill two sets of 50 mL conical tubes with Buffer A and keep them on ice.

**CRITICAL:** Animal work can only be performed after appropriate approval is obtained and under the applicable guidelines and regulations.

### Institutional permissions

All experiments were performed by guidelines from the Institutional Animal Care and Use Committee at Brigham and Women’s Hospital.

### Induction and scoring of DSS-induced colitis in mice

**Timing: 8 days**

8. Add 7.5 g of DSS to 250 mL of autoclaved drinking water for a final concentration of 3% (w/v).
  a. Give control mice autoclaved drinking water without DSS.
  b. Do not allow animals access to any other water source during the seven days.

9. Weigh mice daily.
  a. Weight loss greater than 25% of initial weight is considered an endpoint.
  b. Score each mouse daily according to the following criteria during the DSS treatment period to evaluate the clinical severity of colitis.
    ***Note:*** The disease activity index (DAI) is the combined score of three parameters: weight loss compared to initial weight, the presence of blood in stool, and stool consistency.
    i. Weight Loss.

**Table:** 

Score	Level of weight loss
0	< 1%
1	1–5%
2	5–10%
3	10–15%
4	>15%
    ii. Bleeding.

**Table:** 

Score	Level of bleeding
0	Normal (hemoccult negative, no visible blood in stool)
1	Hemoccult positive (hemoccult positive, no visible blood in stool)
2	Slightly visible blood in the stool (hemoccult positive, visible blood in stool with reddish hue upon smear)
3	Visible blood in the stool (hemoccult positive, obvious blood in stool, but no incrustation around the anus)
4	Gross bleeding (fresh extensive blood around the anus or encrusted on fur)
    iii. Stool Consistency.

**Table:** 

Score	Level of stool consistency
0	Normal (well-formed pellet, solid)
1	Soft (well-formed pellet, soft); 2: Pasty (semi-formed pellet, readily becomes paste upon handling)
2	Pasty (semi-formed pellet, readily becomes paste upon handling)
3	Loose (poorly formed pellet, readily becomes paste upon handling)
4	Diarrhea (no pellet formation, and/or liquid stools)

10. After 7 days, give all animals access to normal drinking water until humane euthanization is performed on Day 8.
11. Weigh mice at the time of sacrifice and proceed with isolation of DCs and CD4^+^ T cells from the lamina propria (LP) of colitic mice for analysis of Smad proteins.

## Key resources table

**Table:** 

REAGENT or RESOURCE	SOURCE	IDENTIFIER
**Antibodies**		

Zombie Aqua Fixable Viability Kit (Dilute 1:1000)	BioLegend	Cat#423101
TruStain FcX (anti-mouse CD16/32) (Dilute 1:25)	BioLegend	Cat#101319; RRID: AB_1574973
7-AAD Viability Staining Solution (Dilute 1:20)	BioLegend	CAT#420403
anti-mouse CD4/FITC (GK1.5) (Dilute 1:100)	BioLegend	Cat#100405; RRID: AB_312690
anti-mouse CD45/BV421 (30-F11) (Dilute 1:100)	BioLegend	Cat#103133; RRID: AB_10899570
anti-mouse CD11c/APC (N418) (Dilute 1:100)	BioLegend	Cat#117310; RRID: AB_313779
anti-mouse I-Ab/PE (AF6–120) (Dilute 1:100)	BioLegend	Cat#116408; RRID: AB_313727
anti-mouse Phospho-Smad2/3 (Dilute 1:50)	Cell Signaling Technology	Cat#8828S; RRID: AB_2631089
Anti-Smad7 (Dilute 1:500)	R&D Systems	Cat#MAB2029
anti-GAPDH (14C10) Rabbit mAb (Dilute 1:5000)	Cell Signaling Technology	Cat#2118
anti-α-Tubulin (11H10) Rabbit mAb (Dilute 1:5000)	Cell Signaling Technology	Cat#2125

**Chemicals, peptides, and recombinant proteins**		

Dextran Sulfate Sodium Salt, Colitis Grade (36,000–50,000 MW)	MP Biomedicals	Cat#9011–18–1
Dulbecco’s Phosphate Buffered Saline (1× PBS)	Thermo Fisher	Cat#14190-144
Dithiothreitol (DTT)	Sigma-Aldrich	Cat#10197777001
Heat-inactivated Fetal Calf Serum (FCS)	Thermo Fisher	Cat#A38402-02
1× Hank’s Buffered Salt Solution (HBSS)	Thermo Fisher	Cat#14175095
HEPES	Thermo Fisher	Cat#15630-080
UltraPure^TM^ 0.5M EDTA, pH 8.0	Thermo Fisher	Cat#15575-038
RIPA buffer	Sigma-Aldrich	Cat#R0278
Tween-20	Sigma-Aldrich	Cat#P1379-500mL
Sodium phosphate dibasic (Na_2_HPO_4_)	Sigma-Aldrich	Cat#S0876-5KG
Potassium phosphate dibasic (KH_2_PO_4_)	Sigma-Aldrich	Cat#P8281-500G
Blotting-Grade Blocker Nonfat dry milk	Bio-Rad	Cat#170-6404
X-VIVO 15 Serum-Free medium with Gentamicin and Phenol Red	Lonza	Cat#04-418Q
Deoxyribonuclease I (DNase I)	Sigma-Aldrich	Cat#DN25-1G
Liberase TL	Sigma-Aldrich	Cat#5401020001
Pierce™ Coomassie (Bradford) Protein Assay Kit	Thermo Fisher	Cat#23200

**Experimental models: Organisms/strains**		

Mouse: C57BL/6J, 8-weeks old, both male and female	Jackson Laboratory	Stock#000664

**Other**		

100 × 15 mm style petri dishes	Falcon	Cat#351029
Sterile 50 mL centrifuge tubes	Olympus Plastics	Cat#28-108
Sterile 25cm^2^ vented cap, TC treated tissue culture flasks	Fisherbrand	Cat#FB012935
2 mL Natural Microcentrifuge Tubes	Seal-Rite	Cat#1620-2700
Corning Falcon Round-Bottom Polystyrene Test Tubes with Cell Strainer Snap Cap, 5 mL	Fisherbrand	Cat#08-771-23
4–20% Mini-PROTEAN® TGX™ Precast Protein Gels	Bio-Rad	Cat#4561096
G:BOX Chemi XX6/XX9 – High-resolution gel imaging system	Syngene	Cat#GBOXCHEMIXX9F08A
BD FACSAria IIu	BD Biosciences	

## Materials and equipment

• Deoxyribonuclease I (DNase I): Reconstitute 100mg in 50 mL sterile H_2_O (2 mg/mL). Store in 1 mL aliquots at −20°C for up to six months.
• Liberase TL: Reconstitute 5 mg with 2 mL 1× PBS (2.5 mg/mL). Store in 1 mL aliquots at −20°C for up to six months.
• Buffer A: 500 mL HBSS; 25 mL FCS; 12.5 mL 1M HEPES (store at 4°C for up to 6 months).
• Buffer B: 500 mL HBSS; 2 mL 0.5M EDTA; 12.5 mL 1M HEPES (store at 4°C for up to 6 months).
• FACS Buffer: 1000 mL (1×) PBS with 20 mL (2%) of fetal calf serum (store at 4°C for up to 6 months).
• Enzyme solution: 1.25 mL of liberase TL (2.5 mg/mL) and 1 mL DNase I (2 mg/mL) to 100 mL of pre-warmed X-VIVO 15 media (Store at 37°C and use the same day).

**Table:** Buffer C

Reagent	Amount	Final concentration
1× Hank’s Buffered Salt Solution (HBSS)	437.5 mL	N/A
1M HEPES	7.5 mL	3.75 mM
0.5M EDTA	5 mL	1.25 mM
Heat-inactivated Fetal Calf Serum (FCS)	50 mL	10%
Dithiothreitol (DTT) (add on the day of the experiment)	0.077 g	1 mM
**Total**	**500 mL**	

Store at 4°C for no more than 24 h.

**Table:** Tris Buffered Saline (TBS) and Tris buffered saline with 0.1% Tween 20 (TBST)

Reagent	Amount	Final concentration
Tris-HCl pH 7.4	24g	20 mM
Sodium Chloride	88g	150 mM
Distilled Water	1000 mL	
**Total**	**1 L**	**10×**
Add 100 mL of 10× TBS to 900 mL H_2_O to make 1× TBS	1 L	1×
Add Tween-20 to 1× TBS to make TBST	1 mL	0.1%
**Total**	**1001 mL**	**1×**

Store at 25°C for up to 1 year.

## Step-by-step method details

### Colonic lamina propria mononuclear cell isolation

**Timing: 6 h**

This step describes dissection of the colon from colitic mice and the isolation of cells from the lamina propria, a layer of connective tissue that houses many of the gut’s immune cells. This layer will be digested, and the cells isolated using enzymatic digestion.

1. Dissect the entire length of the colon by making an incision at the anus and another immediately below the cecum. Remove the colon and wash it by briefly submerging it in 1× PBS to remove any excess blood.
2. Lay the colon flat on a paper towel and remove fecal contents by pushing them out using forceps.
  a. Wet the forceps to make it easier to handle the colon and squeeze out the fecal contents.
  b. Clean the forceps after every sample.
3. Remove as much fat attached to the colon as possible with forceps.
4. Cut the colon vertically and fold open the tissue to expose the lumen.
5. Transfer the colon into a 50 mL conical tube containing ice-cold Buffer A.
6. Vortex the tube for 10 s and then store it on ice.
7. Once all colons are collected and stored in the 50 mL conical tube containing Buffer A, add 10 mL of the pre-warmed Buffer C containing DTT to a 25 cm^2^ flask (one per tissue).
8. Place a metal tea strainer on a large beaker. Briefly, vortex each 50 mL tube with colon tissue in Buffer A and then pour it out over the strainer to remove the buffer.
9. Lay the colon tissue flat on a paper towel for a few seconds to remove any remaining buffer.
10. Transfer the colon tissue to the flask containing 7.5 mL of the pre-warmed Buffer C with DTT.

**CRITICAL:** For multiple samples, work quickly to minimize the time difference between samples and to avoid longer digestions for earlier samples.

11. Firmly close the flasks now containing the tissue with caps and cover them tightly with parafilm to prevent leakage.
12. Horizontally place the sealed flasks with the colons into a 37°C incubating shaker immediately and secure them using tape to prevent them from falling.
13. Incubate for 20 min in the incubator at a shaking speed of 180 rpm.
  a. If the incubator isn’t at 37°C prior to adding samples, add 5 extra minutes to each incubation.
14. During the incubation, thaw the Liberase and DNase I aliquots at 37°C. Once thawed, prepare 10 mL per mouse of the enzyme solution (scale up as needed). Keep the enzyme solution at 37°C.
15. When the incubation is over, place the flasks on ice immediately to stop the reaction.
16. Lay the colon tissue on a paper towel for a few seconds to remove buffer.
17. Transfer the colon tissue to another 50 mL conical tube containing 10 mL of ice-cold Buffer A.
18. Vortex briefly, then pour buffer and colon over the tea strainer.
19. Transfer the colon tissue to a 2 mL Eppendorf tube.
20. Chop each tissue with a pair of clean scissors for 1.5 min.
  a. When chopping the colonic tissue, take care not to scratch the bottom or sides of the tube as the small tissue pieces may get stuck.
  b. Wipe scissors between samples with paper towels and clean buffer.
21. Add 900 μL of cold Buffer B to each tube. Dip the scissors in the suspension to remove any remaining tissue pieces. Keep on ice until all the samples are processed.
  a. Once chopping is done for each sample, finger-flick the tube and check if any large pieces of tissue remain. If so, chop the tissue for another minute or two.
22. Spin down the Eppendorf tubes with tissue at 2,800 × *g* for 1 min at 4°C in a microcentrifuge.
23. Using a P1000 pipette, slowly remove the supernatant and the fat floating at the top as much as possible. Go in a circular motion from the top to bottom.
24. Resuspend the pellet with 1 mL of the pre-warmed Enzyme Solution. Close the lid firmly, vortex to disrupt the pellet, and carefully pour the suspension into a new 7.5 mL flask.
  a. Repeat this step three times with a total of 3 mL of the Enzyme Solution (X-VIVO 15 with liberase and Dnase I) per tissue.
25. Add 7 mL of the pre-warmed Enzyme Solution to each flask to bring up the final volume to 10 mL per tissue sample.
26. Close the tubes firmly and cover the caps tightly with parafilm.
27. Place the flasks in the incubator shaker horizontally and secure them using tape.
28. Incubate at 37 °C at 120 rpm for 40 min.
29. In the last 5 min of the incubation, meanwhile, add 10 mL of ice-cold Buffer A into a separate 50 mL tube with a 100 μm cell strainer on top. Keep on ice.
30. Once the incubation is finished, immediately place flasks on ice to stop the enzymatic reaction.
31. Using a 10 mL pipette, transfer and pass the suspension through the 100 μm cell strainer tube.
32. Wash each flask with 10 mL of ice-cold Buffer A to catch any remaining tissue and transfer it over the corresponding cell strainer/tube for each tissue.
33. Using the plunger from a 1 mL syringe, mash the colon tissue over the 100 μm cell strainer until it is passed through.
  a. You may still see some white membranous tissue left on the cell strainer, but this can be discarded.
34. Add 10 mL of ice-cold Buffer A to wash each cell strainer/tube. Discard the cell strainer after use.
35. Return the top to the 50 mL tube to seal and spin down the samples for 5 min at 500 × *g* at 4°C in a table-top centrifuge.
36. Discard the supernatant in one quick motion, and resuspend the pellet with 10 mL of ice-cold Buffer A.
37. Pipet up and down using a 10 mL pipette and transfer the suspension over a 50 mL conical tube with a 40 μm cell strainer.
38. Wash each cell strainer with 10 mL of ice-cold Buffer A. Bring the final volume up to 30 mL with ice-cold Buffer A.
39. Spin down the samples for 5 min at 500 × *g* at 4°C in a table-top centrifuge.
40. Aspirate the supernatant and proceed with the pellet to the next steps for staining with antibodies and sorting for DCs and CD4^+^ T cells by FACS. Troubleshooting.

### Flow cytometric cell sorting for colonic lamina propria DCs and CD4^+^ T cells

**Timing: 1 h for staining, 1–2 h for sorting (dependent on the number of samples)**

In this step, isolated mononuclear cells from the colonic lamina propria are stained with appropriate antibodies to identify DCs and CD4+ T cells and then sorted via flow cytometry. For more information regarding the theory behind flow cytometry, please refer to Adan et al.^5^

41. Resuspend the above-mentioned pellet in a 50 mL conical tube with 250 μL FACS buffer.

**CRITICAL:** Keep samples on ice to maximize the viability of the cells.

**Figure 1:**
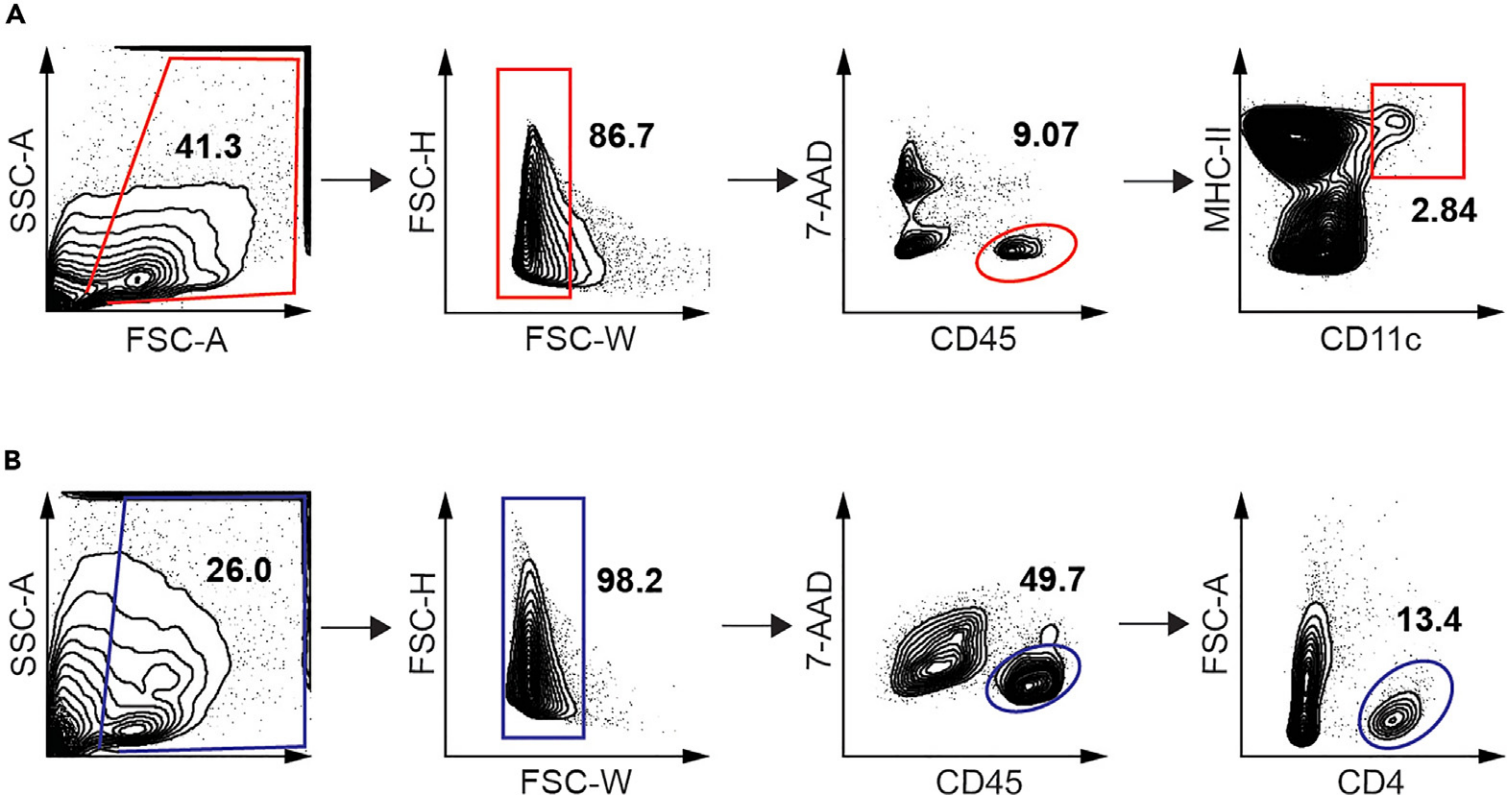
Fluorescence-activated cell sorting (FACS) gating strategy for the isolation of DCs and CD4^+^ T cells from LP by FACS (A) Gated sorting strategy for DCs. Total cells → singlets → Live/Dead^-^ CD45^+^→ CD11c^+^ MHCII^+^. (B) Gating strategy for CD4^+^ T cells. Total cells → singlets → Live/Dead^-^ CD45^+^ →FSC-A^-^ CD4^+^. Numbers listed are frequencies of parent populations.

***Note:*** Sort using a 100 μm nozzle, if possible, to avoid sample clogging during the sort.

42. Add 10 μL of FcR Block Reagent (TruStain FcX) to each sample. Incubate at 4°C for 10 min.
43. Add 2.5 μL of each antibody (CD4 – FITC, CD45 – BV421, I-Ab – PE, CD11c – APC) to the cells in the 50 mL conical tube to equal a dilution of 1:100 along with 12.5 μL of 7-AAD for a 1:20 dilution. Pipet up and down to mix. Incubate at 4°C for 30 min in the dark.
  a. The staining can also be done at RT for 5 min for FcR block and 15 min for antibodies.
  b. Be sure to also prepare fluorescence minus one (FMO) controls for each fluorochrome/antibody to ensure proper gating during the sorting.
44. To wash the samples, add 1 mL of FACS buffer to each sample in 50 mL conical tube, pipet up and down to mix.
45. Spin down 5 min at 500 × *g* at 4°C.
46. Aspirate the supernatant and resuspend the pellet in 1 mL of the FACS buffer.
47. Pass the suspension through a Corning cell strainer snap cap on 5 mL polystyrene round-bottom tube and place them on ice.
48. Sort for CD11c+ DCs (CD45^+^I-Ab^+^CD11c^+^) and CD4+ T cells (CD45^+^CD4^+^) from each sample using a FACSAria IIu flow cytometer (BD Biosciences) (see Figure 1 for gating strategy).

49. Once the cells are sorted, spin down at 500 × *g* for 5 min at 4°C.
50. Remove supernatant. Resuspend cells in FACS buffer and/or Radioimmuno precipitation assay (RIPA) buffer and proceed with intracellular staining and western blotting for phosphorylated Smad2/3 and Smad7.

***Note:*** Smad proteins can also be detected in DCs and CD4+ T cells isolated from other organs such as the mesenteric lymph nodes (MLN) and the spleen from colitic and non-colitic naive mice. If using bone marrow-derived DCs or ex vivo-isolated DCs from spleen or LN and CD4+ T cells from naïve mice, stimulate these cells with TGF-β for at least 30 min to 1 h. CD4+ T cells should be cultured with anti-CD3 and anti-CD28 antibodies for at least 12 h before adding TGF-β. Please note that as of this writing, no commercially available flow antibody is available for Smad7. Troubleshooting.

### Intracellular staining for phosphorylated Smad2/3

**Timing: 3 h**

This step describes the intracellular staining protocol for Smad2/3 via flow cytometry.

**Figure 2:**
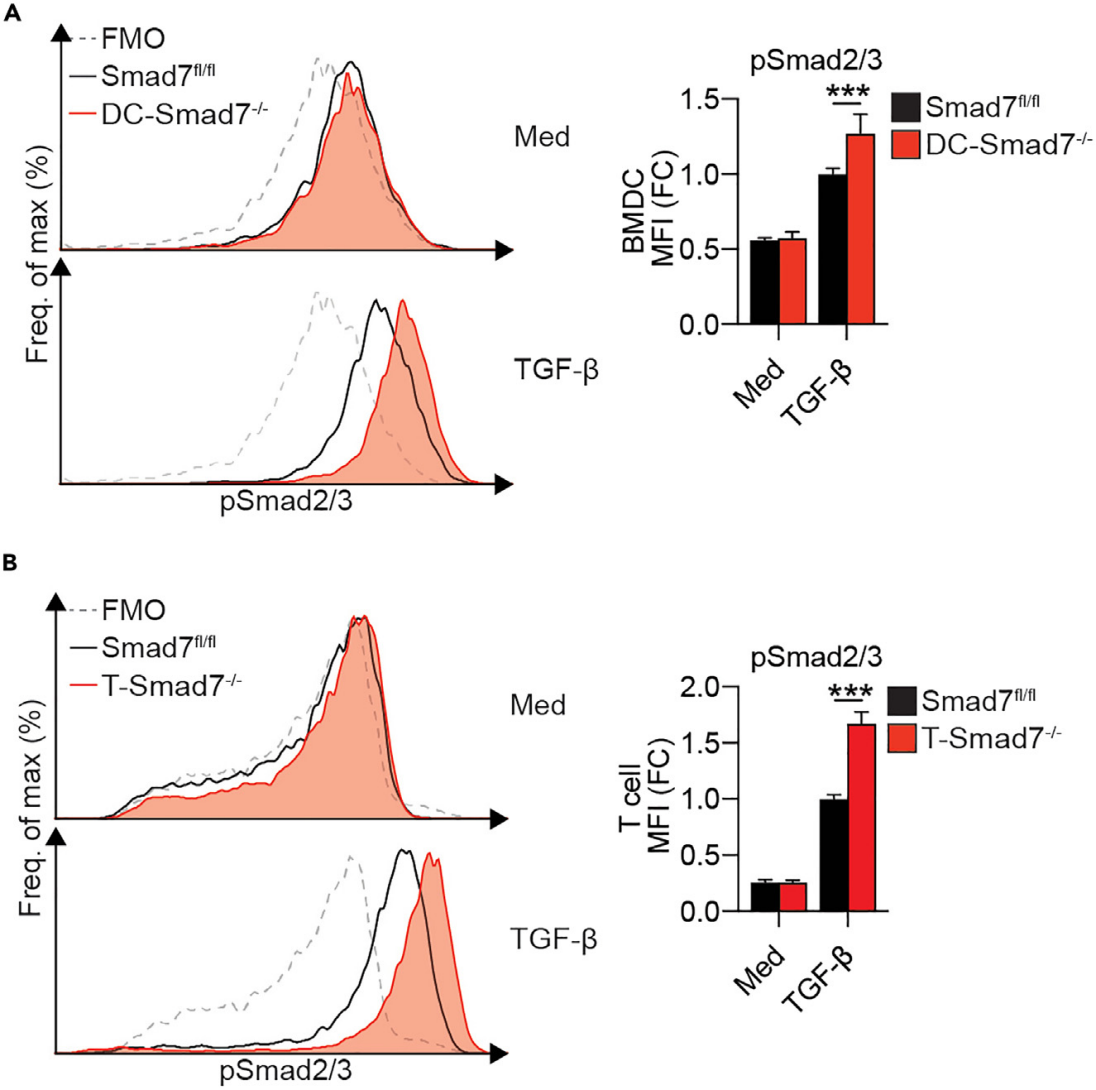
Flow cytometric analysis of phospho-Smad2/3 in DCs and CD4^+^ T cells TGF−β induces Smad2/3 phosphorylation and deletion of Smad7 enhances TGF-β-induced phospho-Smad2/3. (A) Representative FACS histograms (left) and median fluorescence intensities (MFI)s (right) of phospho- Smad2/3 in bone marrow-derived DCs (BMDCs) stimulated with or without TGF-β (2 ng/mL), for 1 h (n = 6). Adapted from reference.^1^ (B) Representative FACS histograms (left) and MFIs (right) of phospho- Smad2/3 in naive CD4^+^ T cells from Smad7^fl/fl^ and T cell specific-Smad7^−/−^ mice stimulated with or without TGF-β (2.5 ng/mL), for 1 h (n = 4). Data representative of ≥3 independent experiments. Means ± SEMs. ∗∗∗p < 0.001 by unpaired t test. Adapted from reference.^1^

***Note:*** Keep cells on ice and protected from light until sorting begins. Troubleshooting.

51. Transfer cells to a 96-well round bottom plate to allow for effective pelleting. Centrifuge for 5 min at 420 × *g*. Discard supernatant.
52. Perform live/dead staining by resuspending the cells in 100 μL of 1× fixable LIVE/DEAD stain (Zombie Aqua Fixable Viability Dye) prepared by diluting 1 μL dye in 1000 μL 1× PBS. Incubate for 10 min at 37°C.
53. Wash cells by adding 100 μL of PBS. Centrifuge for 5 min at 420 × *g*. Discard supernatant.
54. Fix cells by resuspending in 100 μL 2% paraformaldehyde (PFA) in PBS. Incubate at 37°C for 10 min. Centrifuge for 5 min at 420 × *g*. Discard supernatant.
55. Resuspend cells in 100 μL of ice-cold methanol. Incubate at 4°C for 60 min in the dark.
56. Wash cells by adding 200 μL FACS buffer. Centrifuge for 5 min at 420 × *g*. Discard supernatant.
57. Wash cells again by adding 200 μL FACS buffer. Centrifuge for 5 min at 420 × *g*. Discard supernatant.
58. Perform intracellular staining by resuspending cells in 100 μL FACS buffer containing 2 μL of phospho-Smad2/3 antibody. Incubate for 60 min at RT.
59. Wash cells by adding 100 μL FACS buffer. Centrifuge for 5 min at 420 × *g*. Discard supernatant.
60. Wash cells again by adding 200 μL FACS buffer. Centrifuge for 5 min at 420 × *g*. Discard supernatant.
61. Resuspend cells in 200 μL FACS Buffer. Transfer to tubes compatible with your flow cytometer’s sample injection port.
62. Store at 4°C until samples are acquired on a flow cytometer. See Figure 2 for representative flow cytometry data.

### Western blotting analysis of Smad7 in DCs and T cells from colitic mice

**Timing: 2 days**

This step describes how western blotting was used to analyze Smad7 protein expression in FACSorted DCs and T cells from colonic lamina propria.

63. Following initial FACS sorting from the Lamina Propria or following subsequent treatment with TGF-β in culture, collect cells in a tube and centrifuge at 1,000 × *g* for 5 min at 4°C.

**CRITICAL:** Protein degradation is maximum at room temperature. Thus, samples should be kept on ice during processing and at −80°C for storage.

**Pause point:** The cell pellet can be stored at −80°C for several weeks.

64. Wash the pellet with PBS.
65. Remove supernatant and add 200 μL RIPA lysis buffer containing 1× complete mini proteinase inhibitor cocktail to the cells per 10^6^ cells.
66. Incubate cell lysate on ice for 1 h with vortexing every 15 min.
67. Pass through a bent 27-gauge needle 10 times.
68. Sonicate for 10 cycles at 40% amplitude with 1-s pulse and 10 s off on the ice.
69. Centrifuge at 20,000 × *g* for 30 min and collect supernatant in a fresh microfuge tube.

***Note:*** This will be the whole cell lysate.

70. Using the Bradford Protein Assay Kit, measure the protein concentration according to the manufacturer’s instructions (https://www.thermofisher.com/document-connect/document-connect.html?url=https://assets.thermofisher.com/TFS-Assets%2FLSG%2Fmanuals%2FMAN0011181_Coomassie_Bradford_Protein_Asy_UG.pdf) . Adjust protein concentration to 5 μg/μL with the RIPA lysis buffer. Store samples at −80°C.

***Note:*** The RIPA buffer should be used for both the blank and the standard for protein estimation.

**Pause point:** The total protein can be stored at −80°C for several weeks.

**Figure 3:**
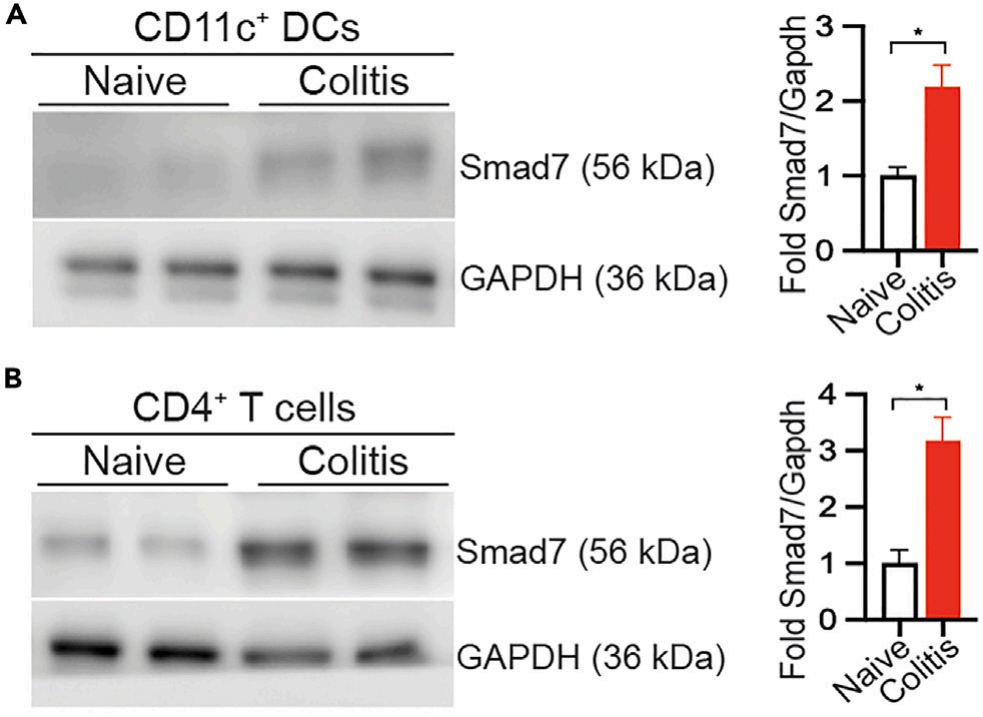
Western blotting analysis of Smad7 in DCs and CD4^+^ T cells Smad7 is increased in (A) CD11c^+^ DCs and (B) CD4^+^ T cells during DSS-induced colitis. Western blot of Smad7 in CD11c^+^ DCs and CD4^+^ T cells isolated *ex vivo* from mesenteric lymph nodes (MLN) of naïve and colitic WT mice after 7 days 3% DSS in drinking water. Western blots were quantitated using the quantitation software Genetools from Syngene. Briefly, the intensity of the blots was measured by adding a box of equal area to cover individual blots, and the equal box was used for the background subtraction. Smad7 intensities were normalized to its respective Gapdh, and fold change was calculated with respect to naïve DCs. Data represent mean ± SD. ∗ represents p < 0.05. Adapted from reference.^1^

***Note:*** Please note that as of this writing, no commercially available flow antibody is available for Smad7. Troubleshooting.

71. Take 30–50 μg total protein and add 6 × loading buffer. Make up the volume to 10 μL using lysis buffer.
72. Heat the samples at 100°C for 10 min and keep them on ice immediately.
73. Centrifuge at 7,000 × *g* for 10 min at RT.
74. Load 10 μL of the protein sample per lane on a gradient of 4%–20% sodium dodecyl sulfate-polyacrylamide gel electrophoresis (SDS-PAGE) gel.
75. Load 10 μL for pre-stained protein standard as a marker.Run a the SDS-PAGE gel at 100 V until the markers are separated.
76. Immerse a PVDF membrane for 20 s in methanol during the SDS-PAGE and incubate in the transfer buffer.
77. Immerse blotting papers in the transfer buffer for 10 min or more.
78. Take out the gel and immerse it in the transfer buffer.
79. Rock gently for 5–10 min.
80. Place the PVDF membrane gently on the gel. Then, place blotting papers on both sides of the PVDF membrane and the gel. Carefully remove bubbles using a roller.
81. Using the wet transfer apparatus, transfer proteins onto the PVDF membrane at 300 mA for 60 min.
82. Wash PVDF membrane twice in Tris buffered saline with 0.1% Tween 20 (TBST) after the transfer.
83. Use 5% non-fat milk in TBST for blocking at room temperature for 60 min on a rocker.
84. Incubate with Smad7 antibody (1:500 dilution) or GAPDH (1:5000 dilution) or α-tubulin (1:5000 dilution) in 5% non-fat milk in TBST overnight at 4°C.
85. After incubation with the primary antibody, rinse the PVDF membrane twice with PBST. Then, wash the membrane thrice with TBST at room temperature for 10 min on a rocker.
86. Incubate the PVDF membrane with the secondary antibody conjugated with HRP in 5% non-fat milk in TBST (1: 5,000 dilution) at room temperature for 1 h on a rocker.
87. Rinse the PVDF membrane twice with TBST. Then, wash the PVDF membrane thrice by TBST at room temperature for 10 min each.
88. Decant the TBST and add the Luminata HRP reagent. Incubate for 5 min.
89. Image on the G:Box ChemiXX6/XX9 – High-resolution gel imaging system.
90. Export images in 300 dpi tiff format for creating the figures. See Figure 3 for representative western data.

## Expected outcomes

This method provides step-by-step instructions for the analysis of SMAD molecules in the colonic lamina propria of colitic mice. Smad molecules are expected to be increased in both DCs and CD4+ T cells during DSS-induced colitis (Figures 2 and 3).

## Limitations

One limitation of the current protocol is that it has only been optimized for these particular SMAD molecules. Analysis of other SMAD molecules or phopho proteins in a different signaling pathway may require optimization. Additionally, it is imperative that the isolation of the colonic lamina propria cells and subsequent flow staining is performed as quickly as possible to maximize viability. Smad proteins can also be detected in DCs and CD4^+^ T cells isolated from other organs such as the mesenteric lymph nodes (MLN) and the spleen from colitic and non-colitic naive mice. If using bone marrow-derived DCs or ex vivo-isolated DCs from spleen or LN and CD4^+^ T cells from naïve mice, stimulate these cells with TGF-β for at least 30 min to 1 h. CD4+ T cells should be cultured with anti-CD3 and anti-CD28 antibodies for at least 12 h before adding TGF-β.

## Troubleshooting

### Problem 1

Low cell viability after flow staining (related to step 48).

### Potential solution

Work quickly when processing multiple samples to avoid longer digestions for earlier samples. Keep samples on ice to maximize viability.

### Problem 2

Low expression of Smad molecules by flow cytometry (related to step 62).

### Potential solution

Smad molecules are difficult to detect by flow cytometry. Therefore, it is ideal to reserve the brightest available fluorophores for these markers.

### Problem 3

Degradation of Smad molecules (related to step 63).

### Potential solution

Protein degradation is maximum at room temperature. Thus, samples should be kept on ice during processing and at −80°C for storage.

## Resource availability

### Lead contact

Further information and requests for resources and reagents should be directed to and will be fulfilled by the lead contact, Dr. Gopal Murugaiyan (mgopal@rics.bwh.harvard.edu).

### Materials availability

This study did not generate any unique reagents.

## Data Availability

This study did not generate datasets/codes.
